# Transcriptomic and Metabolomic Profiling Reveals That KguR Broadly Impacts the Physiology of Uropathogenic *Escherichia coli* Under *in vivo* Relevant Conditions

**DOI:** 10.3389/fmicb.2021.793391

**Published:** 2021-12-16

**Authors:** Dawei Yang, Fengwei Jiang, Xinxin Huang, Ganwu Li, Wentong Cai

**Affiliations:** ^1^Key Laboratory of Veterinary Public Health of Ministry of Agriculture, State Key Laboratory of Veterinary Biotechnology, Harbin Veterinary Research Institute, Chinese Academy of Agricultural Sciences, Harbin, China; ^2^Technical Centre for Animal, Plant, and Food Inspection and Quarantine of Shanghai Customs, Shanghai, China; ^3^Department of Veterinary Diagnostic and Production Animal Medicine, College of Veterinary Medicine, Iowa State University, Ames, IA, United States

**Keywords:** uropathogenic *Escherichia coli*, two-component signaling system, amino acid metabolism, acid resistance, transcriptomic and metabolomic profiling

## Abstract

Urinary tract infections are primarily caused by uropathogenic *Escherichia coli* (UPEC). In contrast to the intestinal *E. coli* strains that reside in nutrient-rich gut environment, UPEC encounter distinct niches, for instance human urine, which is an oxygen- and nutrient-limited environment. Alpha-ketoglutarate (KG) is an abundant metabolite in renal proximal tubule cells; and previously we showed that two-component signaling system (TCS) KguS/KguR contributes to UPEC colonization of murine urinary tract by promoting the utilization of KG as a carbon source under anaerobic conditions. However, knowledge about the KguR regulon and its impact on UPEC fitness is lacking. In this work, we analyzed transcriptomic and metabolomic changes caused by *kguR* deletion under anaerobiosis when KG is present. Our results indicated that 620 genes were differentially expressed in the Δ*kguR* mutant, as compared to the wild type; of these genes, 513 genes were downregulated and 107 genes were upregulated. Genes with substantial changes in expression involve KG utilization, acid resistance, iron uptake, amino acid metabolism, capsule biosynthesis, sulfur metabolism, among others. In line with the transcriptomics data, several amino acids (glutamate, lysine, etc.) and uridine 5′-diphosphogalactose (involved in capsule biosynthesis) were significantly less abundant in the Δ*kguR* mutant. We then confirmed that the Δ*kguR* mutant, indeed, was more sensitive to acid stress than the wild type, presumably due to downregulation of genes belonging to the glutamate-dependent acid resistance system. Furthermore, using gene expression and electrophoretic mobility shift assays (EMSAs), we demonstrate that KguR autoregulates its own expression by binding to the *kguSR* promoter region. Lastly, we performed a genome-wide search of KguR binding sites, and this search yielded an output of at least 22 potential binding sites. Taken together, our data establish that in the presence of KG, KguR broadly impacts the physiology of UPEC under anaerobiosis. These findings greatly further our understanding of KguS/KguR system as well as UPEC pathobiology.

## Introduction

Urinary tract infection (UTI) is one of the most common infections in humans, which affects millions of people each year, thus representing a serious health concern worldwide (Barber et al., 2013). Uropathogenic *Escherichia coli* (UPEC) is the primary etiological agent of all UTIs (Foxman, 2002). These bacteria are usually ingested through mouth, and can persist in the gastrointestinal tract without causing disease. However, if the periurethral area is contaminated by UPEC, the bacteria can enter the urinary tract and establish infections, such as cystitis, pyelonephritis, and in some cases, urosepsis (Ikaheimo et al., 1994; Kalra and Raizada, 2009; Foxman, 2010).

To establish colonization in the gastrointestinal tract, orally acquired UPEC must withstand the extremely acidic gastric juice as well as the acidity of short chain fatty acids produced by intestinal microbiota (Giannella et al., 1972; Donovan et al., 2013). Additionally, UPEC need to tolerate acidic environments within phago(lyso)somes after phagocytosis by macrophages (Marshansky and Futai, 2008; Dragotakes et al., 2020). *E. coli* possess multiple acid resistance (AR) systems, among which the glutamate-dependent AR system (GDAR) is arguably the most potent one (Lin et al., 1995). GDAR relies on 2 major components, glutamate decarboxylase isoforms GadA or GadB (De Biase et al., 1996) and glutamate/λ-aminobutyrate (GABA) antiporter GadC (Hersh et al., 1996). The working model of GDAR is that when the intracellular pH drops to toxic levels (pH < 4.2), glutamate (net charge 0) is converted to GABA (net charge + 1) by GadA (or GadB), producing CO_2_; and during this process, α-carboxylate on glutamate is replaced by a cytoplasmic proton (Capitani et al., 2003; Richard and Foster, 2004). GABA is further exported by GadC in exchange for a glutamate molecule (Gut et al., 2006). In this manner, intracellular protons are pumped out and stress relieved. GadA along with its regulators GadE/X/W and several stress chaperones are encoded on the acid fitness island (AFI), while GadBC are localized ∼2.1 Mb apart from AFI (Smith et al., 1992). In *E. coli* K12, expression of the GDAR is greatly induced in the stationary phase of growth (De Biase et al., 1999), and other conditions, such as acidic pH, osmolar stress and anaerobiosis, can also stimulate GDAR expression (Castanie-Cornet et al., 1999; Weber et al., 2005; Hayes et al., 2006). Although much is known about the regulation of GDAR in *E. coli* K12, GDAR regulation in UPEC is far less investigated.

Two-component signaling system (TCS), typically composed of a membrane-bound histidine kinase (HK) sensor and a cytoplasmic response regulator (RR), is an important mechanism used by most bacterial pathogens to sense and respond to a variety of signals and stimuli, such as nutrients and small-molecule signals. Recognition of physical or chemical signals by the HK sensor domain usually triggers modulation of HK autophosphorylation activity. The phosphoryl group is then transferred to the RR, which is often a DNA-binding protein that acts to alter gene expression (Hoch, 2000; Stock et al., 2000). Several TCSs were shown to be involved in UPEC pathogenesis. For instance, deletion of QseC, the HK of a well-studied TCS QseC/QseB, led to dysregulated nucleotide, amino acid, and carbon metabolism, and consequently the attenuation of UPEC virulence (Kostakioti et al., 2009; Hadjifrangiskou et al., 2011). Additionally, loss of envelope stress response system *cpxRA* impaired UPEC’s ability to colonize urinary tract and to cause systemic infections, which may be attributed to enhanced sensitivity to complement-mediated killing (Debnath et al., 2013).

Urine in the bladder features high-osmolarity, limited iron, and low oxygen availability, and this environment contains mostly amino acids and small peptides (Brooks and Keevil, 1997; Snyder et al., 2004). Therefore, iron acquisition and utilization of amino acids as well as small peptides are important metabolic traits that determine the success of UPEC infections. The human pyelonephritis isolate CFT073 possesses at least ten ferric-uptake systems. Inactivation of the aerobactin receptor IutA or the yersiniabactin receptor FyuA significantly reduces UPEC’s fitness in a mouse infection model, although functional redundancy exists among several iron uptake systems (Garcia et al., 2011). Likewise, deletion of D-serine catabolism genes *dsd* or peptide import genes *oppA*/*dppA* impairs the colonization of murine urinary tract by UPEC (Roesch et al., 2003; Anfora and Welch, 2006; Alteri et al., 2009). Gluconeogenic substrates, like certain amino acids and peptides, can be degraded to oxaloacetate or pyruvate, which can serve as substrates for tricarboxylic acid (TCA) cycle and gluconeogenesis. Thus, defects in gluconeogenesis (phosphoenolpyruvate carboxykinase gene *pckA*) or TCA cycle (succinate dehydrogenase gene *sdhB*) lead to decreases in UPEC fitness during human UTI (Alteri et al., 2009; Subashchandrabose et al., 2014). Notably, under anaerobic conditions, the oxidative branch of TCA cycle, particularly α-ketoglutarate (KG) dehydrogenase (SucAB) and succinyl-CoA synthetase (SucCD), is repressed (Kim and Gadd, 2008); and our previous work has demonstrated that in response to KG, TCS KguS/KguR activates a putative KG dehydrogenase (encoded by *c5032*-*c5035* in a genomic island) and a succinyl-CoA synthetase (encoded by *c5036*-*c5037* immediately downstream of *c5035*) as well as a KG transporter under anaerobic conditions and contributes to the utilization of KG. Lack of *c5032*-*c5037* dramatically impaired the growth on KG (Cai et al., 2013), thus it is likely that C5032-C5037 substitute for their equivalents in TCA cycle (SucABCD) and that ATP is produced during the conversion of KG into succinate by C5032-C5037. Given that KG is particularly abundant in renal proximal tubule cells, it is not surprising that KguS/KguR is required for wild-type level of UEPC colonization in murine urinary tracts (Cai et al., 2013). Therefore, we reason that the presence of KG under anaerobiosis constitutes *in vivo* relevant conditions for UPEC. Although *c5032*-*c5039* were established as direct targets of KguR, the regulon of KguR under *in vivo* relevant conditions remains undefined; and the overall physiological impact of KguR system remains unknown.

In this work, we used transcriptomics and metabolomics to investigate the role of KguR in UPEC physiology on a global scale. Our data demonstrate that in the presence of KG, KguR broadly impacts cellular physiology, including amino acid metabolism, iron uptake systems and acid resistance. Through a genome-wide identification of KguR binding sites, we further suggest that besides the *c5032*-*c5039* cluster, KguR could directly regulate other targets.

## Materials and Methods

### Bacterial Strains and Culture Conditions

Strains and plasmids used in this study are listed in Supplementary Table 1. Aerobic growth was achieved by shaking in air at 180 rpm at 37°C, and anaerobic growth by incubating in a sealed jar with MGC AnaeroPack pouches (Mitsubishi Gas Chemical Company, Japan). M9(gly) minimal medium contains glycerol (0.25% v/v) as an energy substrate, supplemented with M9 salts, 2 mM MgSO_4_, 0.1 mM CaCl_2_, and 1 mg/ml vitamin B1. Trimethylamine N oxide (TMAO, 20 mM) was added as electron acceptor during anaerobic growth, and KG was present at 20 mM. For genetic manipulations, all *E. coli* strains were grown routinely in lysogenic broth (LB). Selective antibiotics were added when necessary at the following concentrations: ampicillin (Amp), 100 μg ml^–1^; kanamycin (Kan), 50 μg ml^–1^; chloramphenicol (Chl), 25 μg ml^–1^.

### Recombinant DNA Techniques

Polymerase chain reaction (PCR), DNA ligation, electroporation and DNA gel electrophoresis were performed according to Sambrook and Russell (2001) unless otherwise indicated. DNA sequencing services were provided by Comate Bioscience Company (China). All restriction and DNA-modifying enzymes were purchased from New England Biolabs or Thermo Fisher Scientific, and used based on the suppliers’ recommendations. Recombinant plasmids, PCR products, and restriction fragments were purified using MiniBEST DNA Fragment purification kit or MinElute gel extraction kit (Takara) as recommended by the supplier. DNA and amino acid sequence analyses were performed using CloneManager software (Scientific & Educational Software, NC). Chromosomal transcriptional *lacZ* fusion was constructed by homologous recombination of the suicidal plasmid pVIK112 carrying a fragment of complete 3′-region or internal fragment of the *kguR* gene (Cai et al., 2013). All oligonucleotides used are listed in Supplementary Table 2.

### RNA-Seq and Data Analysis

RNA-seq analysis was performed using a standard protocol with minor modifications (Sheehan et al., 2020; Li et al., 2021). Wild-type CFT073 and its Δ*kguR* mutant were grown anaerobically in M9(gly) in the presence of KG. After a growth period of 18 h at 37°C, bacteria were quickly spun down at 10,000 g for 2 min, followed by adding 4 mL of RNALater (Thermo Fisher Scientific) to stabilize the bacterial pellets. Total RNA of each sample was extracted using TRIzol Reagent (Invitrogen), and possible DNA contamination was removed with a TURBO DNA-free kit (Thermo Fisher Scientific). One microgram of high-quality RNA (A260/A280 ratio > 2.0 and RIN value > 7.0) was used for each NextGen sequencing library, which was constructed according to the manufacturer’s protocol (NEBNext Ultra Directional RNA Library Prep Kit for Illumina). Sequencing of the libraries was performed using a 2 × 150 paired-end (PE) configuration on an Illumina HiSeq platform according to the manufacturer’s instructions (Illumina, CA). Reads were processed by Cutadapt (v1.9.1) to remove adapter sequences and to discard reads with quality scores < 20 and reads < 75 nt after trimming. Clean reads were aligned to the CFT073 genome (GenBank accession: NC_004431.1) using bowtie2 (version v2.1.9, standard options). Reads were counted using HTseq (version V 0.6.1). Differential gene expression analysis was then performed using DESeq2 (V1.6.3) with R version 3.3.2 following a standard workflow. All genes with a |log_2_(Fold-change)| > 1 and a Benjamini-Hochberg adjusted *p*-value (*q*-value) < 0.05 were considered differentially expressed (differentially expressed genes, DEGs). Supplementary Table 3 lists all DEGs in the mutant compared to the wild type, and raw data are available at the National Microbiology Data Center (NMDC40014023 to NMDC40014028).

Kyoto Encyclopedia of Genes and Genomes (KEGG) pathway enrichment analysis was performed by assigning KEGG pathways to each DEG. The enrichment factor was calculated as the ratio of the number of DEGs enriched in the pathway to the number of all genes in the background gene set. The top 19 enriched pathways relevant to this study are shown in the figure.

### Metabolomics

Bacterial cultures were prepared as described in the RNA-seq section. Bacterial pellets were frozen in liquid nitrogen for 15 min and stored in −80°C until further metabolites extraction. For metabolite extraction, bacterial pellets were resuspended in prechilled methanol containing 0.1% formic acid, followed by an incubation of 5 min on ice. These solutions were then centrifuged at 15,000 rpm for 15 min at 4°C. The supernatants containing extracted metabolites were harvested and filtered through a 0.22 μm filter, and subsequently subjected to liquid chromatography-mass spectrometry/mass spectrometry (LC-MS/MS) analysis.

LC-MS/MS analyses were performed on a Vanquish UHPLC system (Thermo Fisher Scientific) coupled with an Orbitrap Q Exactive HF-X mass spectrometer (Thermo Fisher Scientific). For chromatographic analysis, a C18 Hyperil Gold reversed-phase column (2.1 mm × 100 mm, 1.9 μm, Thermo Scientific, United States) preheated at 40°C was selected, with a 16-min gradient at a flow rate of 0.2 mL/min. The dual-eluent for the positive polarity mode were 0.1% formic acid in H_2_O (eluent A) and methanol (eluent B), respectively; on the other hand, 5 mM ammonium acetate at pH 9.0 (eluent A) and methanol (eluent B), respectively, for the negative polarity mode. The dual-solvent gradient program was set as follows: 2% B, 1.5 min; 2–100% B, 1.5–12 min; 100% B, 12–14 min; 100–2% B, 14–14.1 min; 2% B, 14.1–16 min. Q Exactive HF-X mass spectrometer was operated in positive/negative polarity mode with spray voltage of 3.2 kV, capillary temperature of 320°C, sheath gas flow rate of 35 arb and aux gas flow rate of 10 arb.

Raw data generated by UHPLC-MS/MS were analyzed by Compound Discoverer 3.0 (CD 3.0, Thermo Fisher) for peak alignment, peak picking, and quantitation of each metabolite. The main parameters were set as follows: retention time tolerance, 0.2 min; actual mass tolerance, 5 ppm; signal intensity tolerance, 30%; signal/noise ratio, 3; and minimum intensity, 100,000. Then, peak intensities were normalized to the total spectral intensity, and the normalized data were used to predict the molecular formula according to additive ions, molecular ion peaks and fragment ions. Finally, peaks were matched against the mzCloud^1^ and ChemSpider^2^ database for accurate qualitative and relative quantitative results (Li et al., 2017).

### RNA Isolation and Quantitative Real-Time Reverse Transcription PCR

RNA isolation was performed as described above, and reverse transcription of RNA was done using a HiScript II 1st Strand cDNA Synthesis Kit (Vazyme, China). Melting curve analyses were performed after each reaction to ensure amplification specificity. Differences (n-fold) in transcripts were calculated using the relative comparison method, and amplification efficacies of each primer set were verified as described by Schmittgen et al. (2000). RNA levels were normalized using the housekeeping gene *rpoB* as an endogenous control (Skyberg et al., 2008). qPCR was performed with an Applied Biosystem Q5 Thermocycler using TB Green™ Premix Ex Taq™II Tli RNaseH Plus (Takara) according to the manufacture’s instructions (Li et al., 2011).

### Acid Resistance Assay

Acid resistance assay was done as previously described (Ma et al., 2003). Briefly, bacteria were grown anaerobically in M9(gly) in the absence or presence of KG to stationary phase, and then the cultures were 1:1,000 diluted into prewarmed HCl-buffered LB (pH 2.5) for 2 h acid treatment. Viable counts were measured at time 0 and 2 h after acid challenge. Survival = (CFU_2 h_/CFU_0 h_) × 100%.

### β-Galactosidase Assay

β-galactosidase assay was performed according to Cai et al. (2013), with minor modifications. Briefly, bacteria were grown in M9(gly) with or without KG, followed by harvesting and washing with PBS, and then bacteria were diluted properly in Z buffer. These cultures were diluted 1:10 in Z buffer and assayed for β-galactosidase activity using ortho-Nitrophenyl-β-galactoside (ONPG) as a substrate.

### Electrophoretic Mobility Shift Assay

Electrophoretic Mobility Shift Assay (EMSA) was performed essentially as the reference (Cai et al., 2013). MBP-KguR-His_6_ fusion protein was expressed on the pMal-c2x vector (NEB) and induced by 1 mM IPTG at 16°C. Proteins were purified to homogeneity using Ni-NTA Spin Columns (Qiagen) and dialyzed against the binding buffer. P_*kguS*_ and P_*c*5038_ probes were PCR amplified and gel purified using OMEGA MicroElute Gel Extracion Kit; and P_*kguS*Δ *BS*_ and P_*c*5038Δ *BS*_ probes were chemically synthesized by Genewiz (China). A probe amplified from the coding sequence of *c5036* was used as a negative control (Cai et al., 2013). EMSAs were performed by adding increasing amounts of purified MBP-KguR-His_6_ fusion protein (0–4 μM) to the DNA probe in the binding buffer (10 mM Tris (pH 7.5), 1 mM EDTA, 1 mM dithiothreitol, 50 mM KCl, 50 mM MgCl_2_, 10 mM acetyl phosphate, 1 μg/mL bovine serum albumin, 10% glycerol) for a 30 min incubation at room temperature. The reaction mixtures were then subjected to electrophoresis on a 6% polyacrylamide gel in 0.5 × TBE buffer (44.5 mM Tris, 44.5 mM boric acid, 1 mM EDTA, pH 8.0) at 100 V for 120 min. The gel was stained in 0.5 × TBE buffer containing the SYBR Gold nucleic acid stain for 15 min, before an image was taken using a ChampGel7000 imager (SAGE, China).

### Statistical Analysis

All binary comparisons were analyzed by a Student’s *t*-test (GraphPad 9.0, Prism). A *P*-value < 0.05 was considered statistically significant.

## Results

### Comparative Transcriptomics Define KguR Regulon Under *in vivo* Relevant Conditions

**FIGURE 1:**
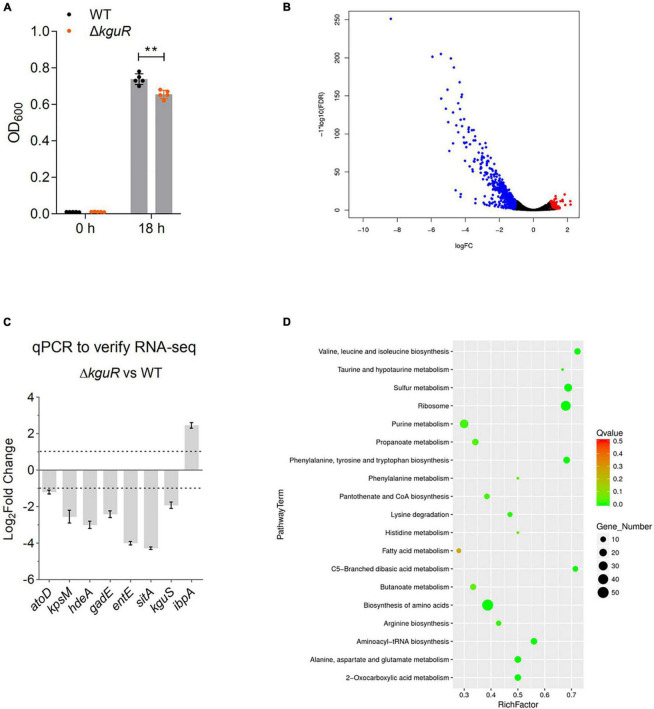
RNA-seq analysis reveals extensive transcriptomic changes due to *kguR* deletion. **(A)** Growth of wild-type CFT073 and the Δ*kguR* mutant. The data represent the mean ± SD of five replicates, and experiments were repeated independently for three times. **(B)** Volcano plot showing transcriptomic changes due to *kguR* deletion. **(C)** qPCR validation of select differentially expressed genes. *atoD* codes for acetate CoA-transferase subunit alpha; *kpsM* for polysialic acid transport protein; *hdeA* for acid stress chaperone; *gadE* for acid resistance transcriptional regulator; *entE* for enterobactin synthase component E; *sitA* for iron/manganese ABC transporter substrate-binding protein; *kguS* for HK of the TCS KguS/KguR; *ibpA* for small heat shock protein. **(D)** A bubble chart demonstrates KEGG pathway enrichment of DEGs. The *y*-axis label represents each pathway, and the *x*-axis indicates the enrichment factor, which was calculated as the ratio of the number of DEGs enriched in the pathway to the number of all genes in the background gene set. The size and color of each bubble represent the number of DEGs enriched in the pathway and the statistical significance of the enrichment, respectively. **P < 0.01 by Student’s *t*-test.

To identify genes whose expression are affected by *kguR* deletion, we first constructed a *kguR* deletion mutant, Δ*kguR*, and assayed the growth kinetics of Δ*kguR* and the wild type (WT) in M9(gly) + KG medium (M9 minimal media containing glycerol as a carbon source, as well as 20 mM KG as an inducing signal) under anaerobiosis. The results showed that Δ*kguR* grew slightly slower than the WT in M9(gly) + KG (Figure 1A), but not in LB rich medium (data not shown), suggesting that KguR is induced and plays a role in the utilization of KG (likely as a carbon and energy source) under these conditions.

Then, we cultured the WT and the Δ*kguR* mutant anaerobically in M9(gly) + KG, and compared their transcriptomes using RNA-seq. With a false discovery rate ≤ 0.05 and fold-change ≥ 2, 620 genes (Figure 1B, volcano plot) were differentially expressed in the Δ*kguR* mutant compared to the WT; of these genes, 513 genes were downregulated and 107 genes were upregulated (A full list of differentially expressed genes is available in Supplementary Table 3).

To validate the RNA-seq data, we performed qPCR on a few representative genes that were differentially expressed in the Δ*kguR* mutant. These genes were selected based on the direction of differential expression (upregulation or downregulation), the extent of the change (2–16-fold), and the relevance to UPEC virulence. The qPCR results are largely consistent with the RNA-seq data (Figure 1C). As expected, target genes encoded on the KG island (except *c5038*) were dramatically downregulated in this experiment, indicating the credibility of this experiment.

All DEGs were then subjected to KEGG pathway enrichment analysis. As shown in Figure 1D, the majority of enriched pathways are related to amino acid metabolism, for example the valine, leucine and isoleucine (branch-chained amino acids, BCAA) biosynthesis. These results indicate that amino acid biosynthesis is likely severely affected due to the loss of *kguR*.

Sixty-four DEGs showed |log2fold−change| ≥ 3, and these were all downregulated in response to the loss of *kguR*. We categorized them into several different functional groups (Table 1), including KG utilization, acid resistance, iron uptake system, amino acid metabolism, capsule biosynthesis, ribosome synthesis, and sulfur metabolism. Each group contains ≥ 4 DEGs, with an average of log_2_fold-change < −3. These results suggest that the abovementioned functions were among the most affected functions of all in the Δ*kguR* mutant.

**TABLE 1:** Information about DEGs with log_2_fold change ≥ 3 in the RNA-seq analysis.

Functional groups	DEGs in the group	Mean log_2_fold change
α-ketoglutarate utilization	7 genes: *kguS c5032*-*c5037 c5039*	–7.99
Acid resistance	8 genes: *hdeABD gadABC gadE mdtE*	–3.9
Iron uptake system	12 genes: *sitABCD entABCDEF chuS hutX*	–3.4
Amino acid metabolism	4 genes: *cysK carA lysA leuA*	–4.1
Capsule biosynthesis	5 genes: *kpsMT* and 3 genes upstream	–4.4
Ribosome synthesis	5 genes: *rplCDWB rpsJ*	–4.5
Sulfur metabolism	6 genes: *cysJIHDNC*	–4.2

### Metabolomic Profiling Reveals Changes in Metabolite Production Due to *kguR* Deletion

**FIGURE 2:**
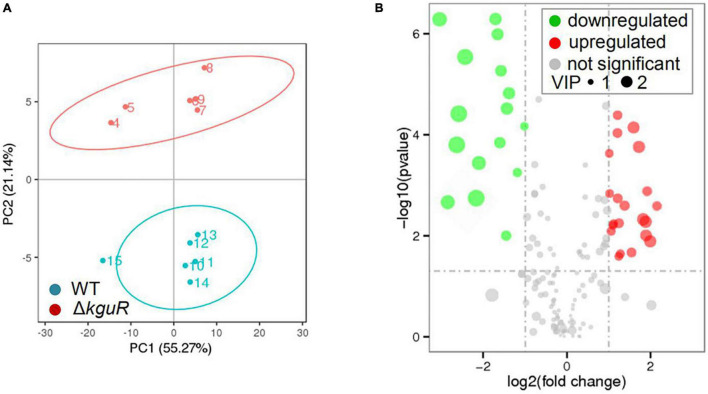
Metabolic profiling of the Δ*kguR* mutant and the WT. **(A)** Principal Component Analysis (PCA) of the WT and Δ*kguR* groups, each containing 6 replicates. Numbers within the circles indicate the replicates in each group. **(B)** Volcano plot showing differentially produced metabolites in the Δ*kguR* mutant compared to the WT. VIP, variable importance in the projection. A metabolite with VIP > 1.0, |log_2_(fold change)| > 1 and *P*-value < 0.05 was considered significant.

To determine metabolic changes caused by *kguR* deletion, such as amino acids, a metabolic profiling was performed on the WT and Δ*kguR* strains using LC-MS/MS (Mitsuwan et al., 2017). A Principal Component Analysis (PCA) was carried out on each group that contains 6 replicates, and the results showed a clear distinction between the WT group and Δ*kguR* group (Figure 2A). In total, 185 metabolites were unambiguously identified. Metabolites in the Δ*kguR* mutant with a *p*-value < 0.05 and an absolute log_2_fold change ≥ 1 relative to the WT were considered significantly differentially produced. A total of 36 metabolites were differentially produced, with 20 being more abundant and 16 less produced in the Δ*kguR* mutant (Figure 2B); and information about these metabolites was listed in Table 2. Several amino acids were downregulated in the Δ*kguR* mutant, including glutamate, threonine, proline, and lysine. Collectively, these data demonstrate that Δ*kguR* mutant has a different metabolic profile than the WT and that amino acid production is severely affected because of *kguR* deletion, which agrees with our KEGG enrichment analysis of DEGs.

**TABLE 2:** Differentially produced metabolites in Δ*kguR* mutant compared to the wild type.

Metabolite	MW^a^	RT (min)^b^	Log2FC^c^	*P*-value	Pathway
**Downregulated**					
1-Amino-1-carboxycyclopropane	101.04774	1.074	–2.63344	0.000158	Cysteine and methionine metabolism
2-O-(6-phosphono-alpha-D-mannosyl)-D-glyceric acid	348.04437	1.097	–2.76081	3.45E-05	Phosphotransferase system (PTS)
2-Tolylbiguanide	185.00913	1.062	–2.43334	2.87E-06	Aromatic compounds metabolism
3-Hydroxy-5-oxohexanoic acid	146.05799	1.731	–1.70719	5.09E-07	Aromatic compounds metabolism
4-Coumaric acid	164.04743	1.602	–1.5766	5.40E-06	Tyrosine and ubiquinone metabolism
4-Hydroxybutyric acid	104.04665	0.973	–1.84029	0.000417	Carbon metabolism
D-(+)-Proline	115.06337	1.104	–1.60292	0.000143	Amino acid metabolism
D-a-Hydroxyglutaric acid	148.03601	0.972	–2.5911	5.78E-05	C5-Branched dibasic acid metabolism
DL-Lysine	146.10559	1.051	–1.0173	6.78E-05	Amino acid metabolism
L-(-)-Threonine	119.05828	1.075	–2.10549	0.000363	Amino acid metabolism
L-Glutamate	147.05323	1.073	–2.58498	3.84E-05	Amino acid metabolism
N-Acetyl-glutamic acid	189.06221	0.977	–1.49233	3.48E-05	Arginine biosynthesis
Phosphorylethanolamine	141.0193	1.047	–1.45249	0.00998	Glycerophospholipid metabolism
Putrescine	88.10005	0.922	–1.18473	0.000559	Amino acid degradation
Succinic acid	118.02671	1.725	–1.65656	1.02E-06	Citric acid cycle
Uridine 5′-diphosphogalactose (UDP-Gal)	566.05108	0.972	–4.57873	2.68E-09	Capsule synthesis
**Upregulated**					
(±)-pantetheine	278.12962	7.214	1.21176	4.10E-05	Pantothenate and CoA biosynthesis
1-(5-Deoxy-5-iodo-beta-D-xylofuranosyl)-2,4(1H,3H)-pyrimidinedione	353.97261	0.924	1.896292	0.000255	Pyrimidine metabolism
2-(N(Omega)-L-arginine)succinic acid	290.1223	1.076	1.0584	0.008108	Arginine/amino acid biosynthesis
2-Aminoethyl 2-hydroxy-3-{[(7E)-1-oxonio-7-tetradecen-1-yl]oxy}propyl phosphate	423.23819	13.448	1.992958	0.012931	Carbon metabolism
2-Aminoethyl 2-hydroxy-3-{[(9E)-1-oxonio-9-hexadecen-1-yl]oxy}propyl phosphate	451.26968	14.06	1.883225	0.005306	Membrane biosynthesis
2-Aminoethyl 2-hydroxy-3-{[(9E)-1-oxonio-9-octadecen-1-yl]oxy}propyl phosphate	479.30116	14.596	1.278359	0.022852	Carbon metabolism
2-Methyl-1,4-benzoquinone	122.0368	12.523	1.890042	0.009855	Quinone metabolism
3-{[(2-Aminoethoxy)(hydroxy)phosphoryl]oxy}-2-hydroxypropyl myristate	425.25395	14.046	1.379168	0.002536	Carbon metabolism
4′-Phosphopantetheine	358.09598	6.211	1.208129	9.21E-05	Pantothenate and CoA biosynthesis
8-{3-Oxo-2-[(2E)-2-penten-1-yl]-1-cyclopenten-1-yl}octanoic acid	292.20101	13.041	1.823651	0.004734	Membrane biosynthesis
Arachidonic acid ethyl ester	338.28157	14.597	1.240199	0.025677	Biosynthesis of unsaturated fatty acids
Dimethirimol	209.15038	1.028	1.214264	0.001821	Pyrimidine metabolism
d-Valerolactam	99.06841	1.018	1.021097	0.001461	Alkaloids and protein metabolism
Glycerophospho-N-palmitoyl ethanolamine	453.28534	14.635	1.10226	0.006247	Membrane biosynthesis
Methyl N-2-acetyl-D-lysinate	202.13172	1.13	1.592648	7.17E-05	Membrane biosynthesis
Mevalonic acid	148.07249	1.17	1.515104	2.87E-05	Secondary metabolite Biosynthesis
Naphthaleneacetamide	185.08419	7.256	1.012378	0.000234	Growth regulators
PHENYLBUTYRIC ACID	164.08382	12.432	1.545144	0.021495	Carbon metabolism
Scoparone	206.05556	8.886	1.11586	0.005771	Phenylpropanoids metabolism
Tetranor-12(S)-HETE	248.175	12.803	1.244274	0.005663	Biosynthesis of unsaturated fatty acids

*^a^MW, molecular weight.*

*^b^RT, retention time.*

*^c^FC, fold change (ΔkguR vs. WT).*

### KguR Promotes Acid Resistance of Uropathogenic *Escherichia coli*

**FIGURE 3:**
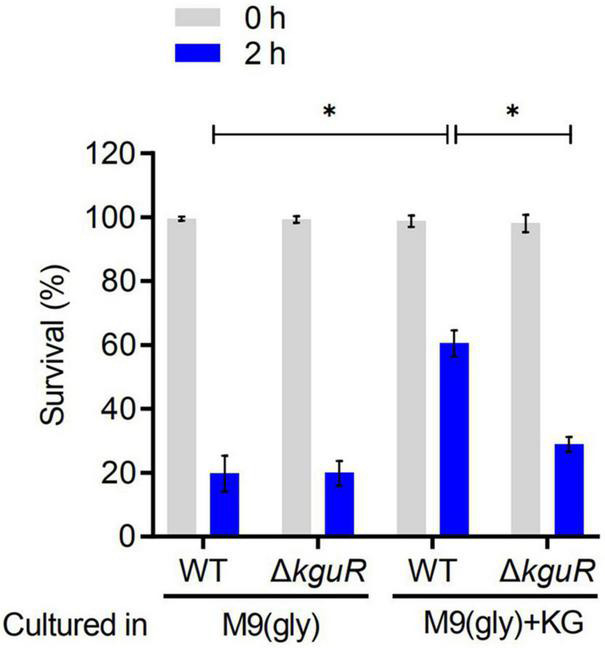
KguR promotes acid resistance of UPEC. Bacteria were grown anaerobically in M9(gly) in the absence or presence of KG to stationary phase, and then the cultures were diluted into acidic LB (pH 2.5) for 2 h acid treatment. Viable counts were measured at time 0 and 2 h after acid challenge. Survival = (CFU_2 h_/CFU_0 h_) × 100%. The data shown are the means ± SEM from three independent experiments. *P < 0.05.

To test whether the loss of *kguR* render UPEC more sensitive to acid treatment, the WT and Δ*kguR* strains were cultured in M9(gly) and M9(gly) + KG, respectively, followed by a treatment with pH 2.5 acidic LB. Figure 3 showed that there was no difference in acid sensitivity between the WT and Δ*kguR* strains when they were both cultured in M9(gly); by contrast, Δ*kguR* was significantly more sensitive to the WT when they were both cultured in M9(gly) + KG. Additionally, wild-type CFT073 grown in the presence of KG was more resistant to acid than those grown in the absence of KG. Together, these data indicate that KguR promotes acid resistance of UPEC, and this may be attributed to the import and conversion of KG into glutamate.

### KguR Autoregulates Its Own Expression in Response to KG

**FIGURE 4:**
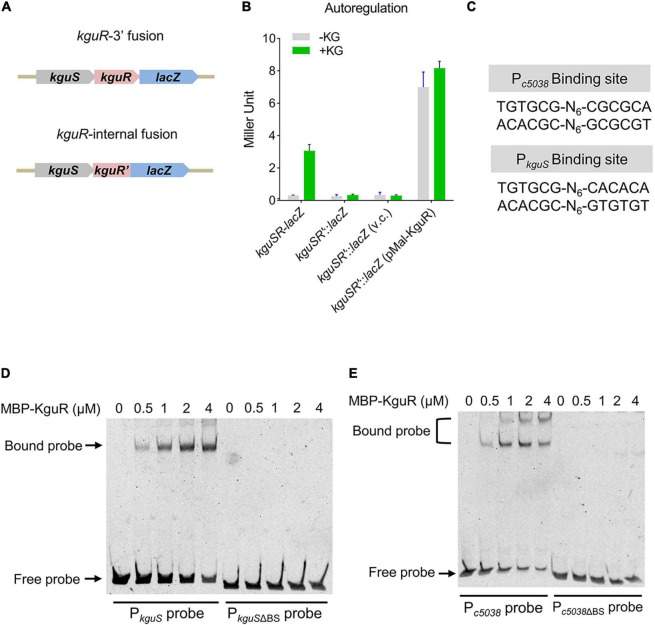
KguR directly autoregulates its own expression. **(A)** A schematic showing the construction of *kguSR*-*lacZ* fusion strain. **(B)** KguR autoregulates its own expression. KG, α-ketoglutarate; and v.c., vector control. The means ± SEM from three independent experiments are presented. **(C)** KguR binding sites in the promoter regions of *c5038* and *kguS*. N stands for random nucleotides between the imperfect inverted repeats. **(D,E)** Binding of KguR protein to native and mutant promoters of *c5038* **(D)** and *kguS* **(E)**. Purified MBP-KguR fusion protein was added in different concentrations in each reaction mixture as indicated. DNA probes were stained with SYBR green nucleic acid stain. BS, binding site.

We have shown that *kguS* and *kguR* co-transcribe, thus forming a transcriptional unit (Cai et al., 2013). RNA-seq analysis revealed that deletion of *kguR* reduced *kguS* expression (Supplementary Table 3), implying that *kguSR* might be autoregulated. To test this, a 5′ chromosomal fusion, in which *lacZ* was placed immediately downstream *kguR*, and an internal fusion, in which *kguR* was fused to *lacZ* but was disrupted by the suicide vector pVIK112, were constructed (Figure 4A and Supplementary Figure 1). As shown in Figure 4B, disruption of *kguR* abolished the *kguSR*-lacZ expression in the presence of KG; and this aligns with the RNA-seq data. Furthermore, expression of plasmid-borne *kguR* in the *kguSR*′-*lacZ* fusion strain dramatically increased the *kguSR*′-lacZ expression. Therefore, these results indicate that KguR autoregulates its own expression.

We then hypothesized that KguR autoregulates itself through directly binding to the *kguSR* promoter. By alignment of the promoter regions of *c5038* and *kguS*, we identified a relatively conserved motif (Figure 4C). Using EMSA, we showed that KguR could indeed bind to the *kguSR* promoter region; whereas it could not bind to a promoter variant lacking the putative binding motif. Similar results were also obtained with the *c5038* promoter region (Figures 4D,E). Altogether, our data demonstrate that KguR directly autoregulates its own expression.

### Genome-Wide Search for KguR Binding Sites

To identify more KguR binding sites and potentially discover additional direct targets of KguR, we carried out a genome-wide search of KguR binding regions in the CFT073 genome using the Pattern Locator program (Mrazek and Xie, 2006) with a TGTG(T/C)G-N_5–15_-C(G/A)C(G/A)CA consensus. Table 3 lists an output of 22 entries, including the binding regions of *c5038* and *kguS*. Among these entries, only 6 are localized in intergenic regions, while others are within coding sequences. Aside from *c5038* and *kguS*, 3 genes containing putative binding sites exhibited gene expression changes in the RNA-seq analysis; and these three genes code for iron transport protein SitA (∼20-fold downregulation), acid phosphatase AppA (∼2.3-fold downregulation), and an aromatic amino acid transaminase (∼2.2-fold downregulation), respectively. Therefore, these results suggest that KguR may associate with additional sites within the genome and directly regulate more target genes, such as *sitA* and *appA*.

**TABLE 3:** List of potential KguR binding sites identified by the Pattern Locator program.

Start	End	Length	Location^a^	Motif^b^	Functional annotation^c^
699341	699361	21	CDS	**TGTGCG**TCAGGTACG**CGCGCA**	Deaminated glutathione amidase
1084525	1084551	27	CDS	**TGTGCG**TGCACCAACCAAGGC**CACGCA**	Histidine-type acid phosphatase AppA
1449366	1449386	21	CDS	**TGTGCG**CCGGTTTCA**CGCGCA**	Iron ABC transporter substrate-binding protein SitA
1567110	1567128	19	CDS	**TGTGTG**AGTTCCT**CGCGCA**	Exoribonuclease YciV
1619855	1619872	18	CDS	**TGTGCG**ATACCG**CGCGCA**	Oxidoreductase ycjS
1624662	1624686	25	CDS	**TGTGTG**CGGGAATGGCCTG**CGCACA**	Porin OmpG
1624664	1624686	23	CDS	**TGTGCG**GGAATGGCCTG**CGCACA**	Porin OmpG
1652910	1652935	26	Intergenic	**TGTGCG**GGCGTTAGTGTCAG**CGCACA**	Metal ABC transporter permease
1778230	1778252	23	Intergenic	**TGTGCG**TAGCCATGCCA**CACACA**	Fimbria periplasmic chaperone
1778230	1778250	21	Intergenic	**TGTGCG**TAGCCATGC**CACACA**	Fimbria periplasmic chaperone
2009374	2009399	26	CDS	**TGTGTG**CAGAACAGTGAAGG**CGCACA**	Bifunctional nicotinamidase/pyrazinamidase PncA
2535426	2535445	20	Intergenic	**TGTGCG**TGAGGCGG**CGCACA**	S-formylglutathione hydrolase YeiG
2552080	2552102	23	CDS	**TGTGTG**TTCACTGGATT**CACGCA**	1-phosphofructokinase FruK
2587706	2587725	20	CDS	**TGTGCG**AACGACAT**CGCGCA**	Ferredoxin-type protein NapG
2608815	2608835	21	CDS	**TGTGCG**AACAACGGG**CGCACA**	Transcriptional regulator AtoC
2722901	2722917	17	CDS	**TGTGCG**GAAGT**CGCACA**	Beta-ketoacyl-ACP synthase I FabB
3337753	3337774	22	CDS	**TGTGCG**CGCCAGGAGA**CGCGCA**	Membrane protein
3928415	3928436	22	CDS	**TGTGCG**GCTAACGCTG**CGCGCA**	Nitrite transporter NirC
4808054	4808078	25	CDS	**TGTGTG**TCGCCGGGCTTAA**CGCGCA**	Aromatic amino acid transaminase
4815816	4815835	20	Intergenic	**TGTGCG**GAAAACCG**CGCACA**	Di-carboxylate transporter C5038
4821809	4821826	18	Intergenic	**TGTGTG**GTTTAT**CGCACA**	Sensor kinase KguS
5003618	5003637	20	CDS	**TGTGCG**GGCTGTGT**CGCGCA**	NAD(P)H-hydrate dehydratase/NAD(P)H-hydrate epimerase

*^a^Indicates the location of a motif, either in the coding sequence (CDS) or the intergenic region.*

*^b^Bold letters represent the relatively conserved binding motif.*

*^c^Indicates the functional annotation of the gene containing the motif or the gene downstream of the motif.*

## Discussion

*E. coli* generally live in the intestinal environment of a host, where nutrients are rich and diverse. By contrast, UPEC can colonize and cause infections in the urinary tract, such as bladder and kidney, which is an oxygen- and nutrient-limited environment (Melican et al., 2008; Wang et al., 2008; Alteri and Mobley, 2015). Previously, we found that KguS/KguR promotes UPEC colonization of murine urinary tracts by activating a cluster of genes involved in the anaerobic utilization of KG, a TCA cycle intermediate and a particularly abundant metabolite in renal proximal tubule cells (Cai et al., 2013). We began this current study aiming to delineate the physiological influence of KguR-mediated KG utilization. A combination of transcriptomics and metabolomics approaches reveals that loss of *kguR* has a broad impact on UPEC physiology, e.g., acid resistance, iron uptake, capsule biosynthesis, and amino acid metabolism. This study, therefore, provides a deeper understanding of role of KguR and its regulon in UPEC pathobiology.

KG can be utilized by UPEC through C5032-C5037 to produce NADH and ATP, facilitating bacterial expansion and subsequent colonization within urinary tract (Cai et al., 2013). KG is also involved in amino acid metabolism, such as oxidative deamination and transamination. For instance, KG lies at the intersection of TCA and amino acid biosynthesis pathways, where KG can be readily converted into glutamate through glutamate synthase or glutamate dehydrogenase (Kim and Gadd, 2008). Indeed, a lack of *kguR* led to reduced production of glutamate as revealed by metabolomics (Table 2). Glutamate is a key metabolite in *E. coli*, providing approximately 85% of organic nitrogen that are incorporated into a variety of amino acids and nucleic acid bases (Huergo and Dixon, 2015). Our metabolic profiling indicates that at least three amino acids, threonine, proline, and lysine, were less produced in the Δ*kguR* mutant compared to the WT. In addition, a great number of genes involved in amino acid biosynthesis, for example branch-chain amino acid biosynthesis, were downregulated (Supplementary Table 3 and Supplementary Figure 2). Given that proper amino acid utilization is a crucial fitness trait for UPEC, we suggest that a lack of *kguR* could affect UPEC growth *in vivo* through amino acid metabolism. KG may also be utilized to synthesize Coenzyme B, and involved in producing 4-hydroxy-2-oxoglutarate by 4-hydroxyglutamate transaminase, which is split into pyruvate and glyoxylate (Watanabe et al., 2012).

UPEC strains may produce two catecholate siderophores, enterobactin, and salmochelin (a glucosylated form of enterobactin) (Henderson et al., 2009). Although most *E. coli* strains can synthesize and utilize enterobactin, this siderophore contributes significantly to UPEC virulence (Johnson et al., 2005). Our RNA-seq data show that at least 6 genes (*entABCDEF*) involved in enterobactin biosynthesis were downregulated > 8-fold (Supplementary Table 3). Biosynthesis of enterobactin requires L-serine, and we found that at least two genes involved in serine utilization were downregulated, i.e., *tdcC* encoding a serine transporter and *serC* encoding a 3-phosphoserine aminotransferase. As a result of reduced expression of these genes, production of enterobactin can be impaired. Furthermore, *sitABCD* encoding an iron transport system and heme utilization genes *hutX* and *chuS* were also downregulated. Together, these results suggest that lacking *kguR* could compromise iron uptake and utilization, thus reducing fitness during UTI.

UPEC encounter both alkaline and acidic environments during colonization in the gastrointestinal tract (de Jonge et al., 2003). Ileum usually has a pH of ∼8–9; and alkaline pH is a stress condition for bacterial survival, but sometimes can be a signal for relevant bacterial behavior, such as flagellar motility (Nhu et al., 2021) and toxin production (Gonzales et al., 2013). In this study, several AR2-related genes including *gadABC* and *hdeAB* were substantially downregulated. *gadE* expression was reduced by ∼8-fold; since GadE is a central regulator governing AR2 expression (Ma et al., 2003), it is likely that KguR affects *gadABC* and *hdeAB* through GadE. Polyamines, such as spermidine and putrescine, can induce the AR2 system in *E. coli* through upregulating *gadE* and *rpoS* (Chattopadhyay and Tabor, 2013). Interestingly, we found that *potD* gene encoding a polyamine ABC transporter substrate-binding protein was downregulated ∼2.5-fold (Supplementary Table 3) and that putrescine production was decreased by ∼2.3-fold in the *kguR* mutant (Table 2). We, thus, suggest a mechanism by which a lack of *kguR* resulted in reduced intracellular polyamine concentration, which led to lessened expression of *gadE* and consequently *gadABC* and *hdeAB*. Therefore, enhanced UPEC fitness *in vivo* by KguR may be partially explained by stronger acid resistance.

Our data showed that *kpsMT* was downregulated ∼16-fold. The *kpsMT* genes, which are localized on the *pheV* genomic island (Lloyd et al., 2007), encode components for a polysialic acid ABC transporter that is responsible for group II capsule biosynthesis. These genes contribute to virulence in UPEC, likely by enhancing adherence to urothelial cells and evasion of phagocytosis by host phagocytes (Foxman et al., 1995; Bliss and Silver, 1996). Additionally, uridine 5′-diphosphogalactose (UDP-Gal), an intermediate providing Gal-1-P for capsule biosynthesis (Drummelsmith and Whitfield, 1999), was 25-fold less abundant in the Δ*kguR* mutant. Together, these results suggest that loss of *kguR* could cause defects in capsule production.

KguR autoregulates itself by binding to the promoter region of *kguSR* (Figure 4). Autoregulation is highly common in signal transduction systems, like TCSs (Groisman, 2016) and extracytoplasmic function sigma factors (Li et al., 2021), because this mechanism can provide a surge of active RR to rapidly carry out the genetic program for adaptation (Groisman, 2016). It is somewhat surprising that only 3 potential binding sites besides those associated with *c5038* and *kguS* were found in intergenic regions, while 16 were found in CDS region. *sitA* contains a binding site in its CDS, and its expression was dramatically reduced by ∼16-fold. Moreover, *sitBCD* genes that cotranscribe with *sitA* were downregulated to similar extent (Table 1). These results imply that KguR might regulate the *sitABCD* operon by directly binding to the *sitA* coding region; however, the precise mechanism warrants further investigation. Experimental approaches are needed to identify KguR binding sites on a genome-wide scale under various conditions. One possibility to explain the difference between the number of KguR regulated genes and the number of KguR regulated promoters would be that KguR indirectly modulates many genes via altered utilization of KG and subsequent amino acid metabolism. These, again, highlight the importance of KguR in modulating UPEC physiology.

## Conclusion

In conclusion, this work provides a comprehensive overview of KguR’s impact on UPEC physiology. Our work provides information about how defects in KG utilization can affect pathobiology. TCSs control virulence traits in many pathogens, and inhibitors of TCSs can reduce virulence without killing the pathogens, thereby imposing little selective pressure on bacteria for drug resistance (Gotoh et al., 2010). A well-studied example is LED209, which inhibits the binding of signals to the HK QseC, leading to the suppression of pathogenicity in enterohemorrhagic *E. coli* (Rasko et al., 2008). Therefore, in-depth understanding of the virulence-associated factor KguS/KguR is indispensable, as it paves the way for discovering antagonists against this TCS, as well as possible treatments for UTI.

## Data Availability Statement

The datasets presented in this study can be found in online repositories. The names of the repository/repositories and accession number(s) can be found below: https://nmdc.cn/resource/search, NMDC40014023 to NMDC40014028.

## Author Contributions

WC and GL conceived, designed the experiments, wrote the manuscript, and provided the resources and the funding. WC, DY, FJ, and XH performed the experiments. WC and DY analyzed the data. All authors participated in the discussion of the results and reviewed the manuscript.

## Conflict of Interest

The authors declare that the research was conducted in the absence of any commercial or financial relationships that could be construed as a potential conflict of interest.

## Publisher’s Note

All claims expressed in this article are solely those of the authors and do not necessarily represent those of their affiliated organizations, or those of the publisher, the editors and the reviewers. Any product that may be evaluated in this article, or claim that may be made by its manufacturer, is not guaranteed or endorsed by the publisher.

## Footnotes

1
https://www.mzcloud.org/

2
http://www.chemspider.com/
