# Supplementary material for: Transcriptomic and Metabolomic Profiling Reveals That KguR Broadly Impacts the Physiology of Uropathogenic Escherichia coli Under in vivo Relevant Conditions
Source: Front Microbiol. 2021 Dec 16;12:793391. doi: 10.3389/fmicb.2021.793391 (PMC8716947; doi:10.3389/fmicb.2021.793391)
Supplement: Supplementary file 1 [file Data_Sheet_1.pdf]

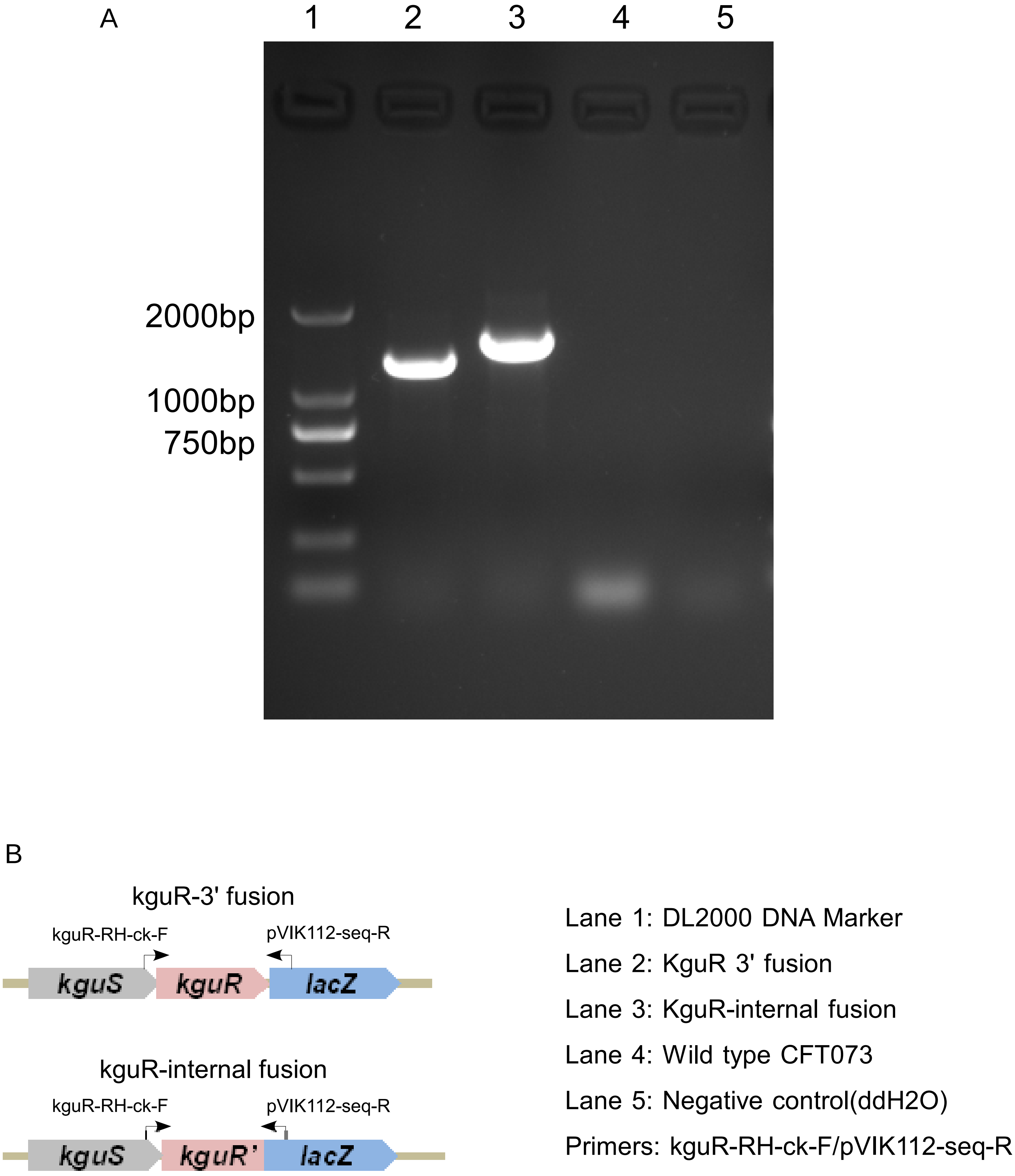

Figure S1. PCR to verify the construction of the *lacZ* fusion strains. **A**. The gel image. The templates used for each lane are shown in the down right region. **B**. A schematic showing gene organization of the constructs as well as the positions for confirmation primers.
