# Supplementary material for: Transcriptomic and Metabolomic Profiling Reveals That KguR Broadly Impacts the Physiology of Uropathogenic Escherichia coli Under in vivo Relevant Conditions
Source: Front Microbiol. 2021 Dec 16;12:793391. doi: 10.3389/fmicb.2021.793391 (PMC8716947; doi:10.3389/fmicb.2021.793391)
Supplement: Supplementary file 2 [file Data_Sheet_2.pdf]

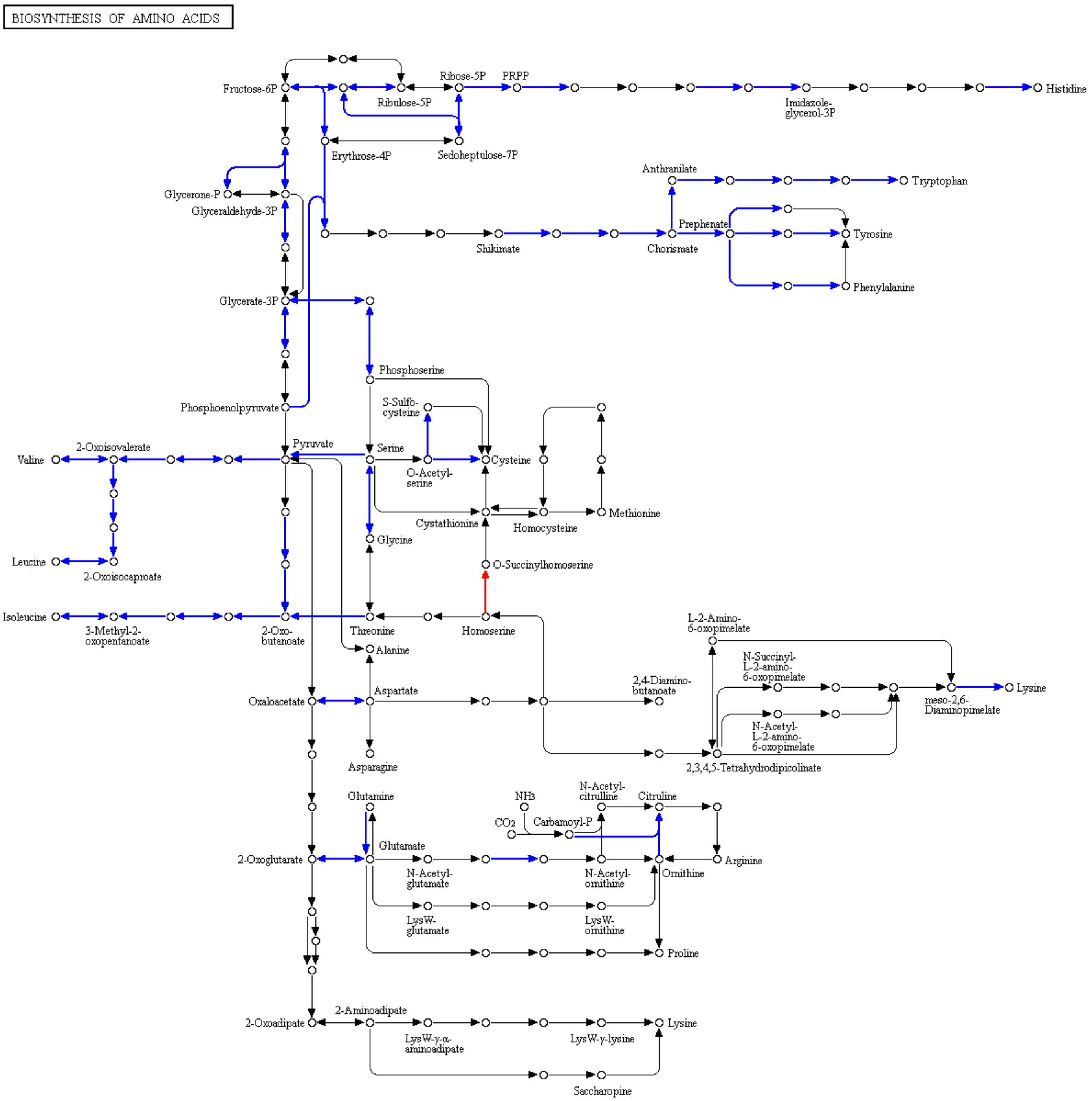

Figure S2. Differentially expressed genes involved in amino acid biosynthesis pathways. Blue arrows indicate downregulation of the genes responsible for that reaction, while red arrows indicate upregulation of the genes responsible for that reaction.
