# Supplementary material for: Transcriptomic and Metabolomic Profiling Reveals That KguR Broadly Impacts the Physiology of Uropathogenic Escherichia coli Under in vivo Relevant Conditions
Source: Front Microbiol. 2021 Dec 16;12:793391. doi: 10.3389/fmicb.2021.793391 (PMC8716947; doi:10.3389/fmicb.2021.793391)
Supplement: Supplementary file 3 [file Table_1.DOCX]

**Table S1. Strains and plasmids.** The genotypes of all *E. coli* strains and information about the plasmids used in this study.

| **Bacterial strains and plasmids** | **Genotype or relevant characteristics** | **Source or Reference** |
| --- | --- | --- |
| ***Bacterial strains*** |  |  |
| DH5α | Plasmid propagation strain | Invitrogen |
| CFT073 | Blood isolate from a patient with acute pyolonephritis | ([1](#_ENREF_1)) |
| LMP10 | CFT073 ∆*lacZYA* | ([2](#_ENREF_2)) |
| Δ*kguR* | Deletion of *kguR* in CFT073 | ([2](#_ENREF_2)) |
| ***Plasmids*** |  |  |
| pMal-c2x | P_tac_ promoter, Ap^R^ | NEB |
| pVIK112 | Ori R6K; RP4 origin of transfer; Km^R^; for *lacZY* fusion | ([2](#_ENREF_2), [3](#_ENREF_3)) |
| pKD3 | template for λ-Red ChlR cassette | ([4](#_ENREF_4)) |
| pCP20 | encodes FLP recombinase for removal of resistance cassette | ([4](#_ENREF_4)) |
| pKD46 | λ-Red recombinase expression | ([4](#_ENREF_4)) |

**References**

1. **Welch RA, Burland V, Plunkett G, 3rd, Redford P, Roesch P, Rasko D, Buckles EL, Liou SR, Boutin A, Hackett J, Stroud D, Mayhew GF, Rose DJ, Zhou S, Schwartz DC, Perna NT, Mobley HL, Donnenberg MS, Blattner FR.** 2002. Extensive mosaic structure revealed by the complete genome sequence of uropathogenic *Escherichia coli*. Proc Natl Acad Sci U S A **99:**17020-17024.

2. **Cai W, Wannemuehler Y, Dell'anna G, Nicholson B, Barbieri NL, Kariyawasam S, Feng Y, Logue CM, Nolan LK, Li G.** 2013. A novel two-component signaling system facilitates uropathogenic Escherichia coli's ability to exploit abundant host metabolites. PLoS Pathog **9:**e1003428.

3. **Kalogeraki VS, Winans SC.** 1997. Suicide plasmids containing promoterless reporter genes can simultaneously disrupt and create fusions to target genes of diverse bacteria. Gene **188:**69-75.

4. **Datsenko KA, Wanner BL.** 2000. One-step inactivation of chromosomal genes in Escherichia coli K-12 using PCR products. Proc Natl Acad Sci U S A **97:**6640-6645.
